# Remote sensing of indoor thermal environment from outside the building through window opening gap by using infrared camera

**DOI:** 10.1016/j.enbuild.2023.112975

**Published:** 2023-05

**Authors:** Xiaomeng Chen, Ziwei Zou, Fulin Hao, Yang Wang, Chuansong Mei, Yuhan Zhou, Da Wang, Xudong Yang

**Affiliations:** a Department of Building Science, School of Architecture, Tsinghua University, Beijing 100084, China; b College of Information and Electrical Engineering, China Agricultural University, Beijing 100084, China; c Shanxi Research Institute for Clean Energy, Tsinghua University, Taiyuan 030032, China; d Beijing District Heating Group, Beijing 100022, China

**Keywords:** Infrared thermography, Remote sensing, Indoor temperature, Nonintrusive technique, Housing temperature survey

## Abstract

Investigation of housing indoor temperature is important for understanding the comfort, health and living conditions of the local residents. The traditional method to measure indoor temperature is to place sensors at the target places, which is not only expensive but also inconvenient for indoor temperature investigation, especially for the investigation at community and city scale. In this study, a novel method was proposed to obtain the indoor temperatures remotely from outside the building through window opening area using and infrared camera. Compared with the traditional contact measurement method, the proposed remote sensing method could detect the indoor temperature without entering the room. Moreover, the infrared image could reflect the spatial distribution information of indoor temperature. To verify the feasibility and accuracy of this method, an experiment was conducted in a test room under heating, transitional, and cooling conditions with various window opening grades. It was found that the infrared images at the window opening area could reflect the spatial distribution of indoor temperature with an accuracy within 0.5 °C under stable heating and transitional conditions. In the fan coil cooling condition, however, although the infrared image can reflect the cold air flow pattern, the deviations between the infrared temperature and the measured room temperature exceeded 1.0 °C. The effect of window opening grade on the recognition accuracy kept within 0.5 °C.

## 1 Introduction

People spend almost 90% of their time indoors. Therefore, the indoor thermal environment is closely related to occupant comfort, well-being, and health [1–3]. Extreme heat and cold exposure poses health risks, particularly for the elderly, children, and those with chronic illness [4–6]. Jevons et al. and Tham et al. investigated the lower and upper indoor temperature thresholds through systematic literature review [7,8]. They concluded that negative health effects in the general population start to occur when the indoor temperature is lower than 18 °C and higher than 26 °C-32 °C. They also reported the insufficient data and heterogeneity of the studies reviewed. It is difficult to obtain indoor environment data since the indoor climates of individual dwellings vary with complex factors, including the outdoor climate, envelope quality, air-conditioning equipment, occupant’s behavior, etc. [9–12].

A common approach to obtain indoor air temperature data is to install thermometers at target places to measure the air temperature directly. Morey et al. conducted a temperature monitoring survey of 122 houses to investigate the extent of overheating risk in summer and found that significant proportions of dwellings were uncomfortably warm in summer, especially in bedrooms and living rooms [13]. The researchers have concluded that the overheating risk will increase owing to climate change, especially for houses with highly insulated envelope [14,15]. A cold indoor environment in winter is also a concern. Many studies have indicated that an indoor environment with a low temperature has a notable impact on the health of vulnerable people. Wang et al. investigated 292 households with children and collected 877 air samples from bedrooms and living rooms. They found a significant correlation between low indoor temperature and the prevalence of the common cold among children [16]. Similarly, Han et al. conducted a prospective cohort study of 285 community-dwelling elderly individuals. They collected indoor temperature and acute respiratory illness incidence data continuously and found a negative correlation between indoor temperature and incidence [17]. Furthermore, Miguel-Bellod et al. reported a relationship between poverty and indoor temperature in winter. They conducted a survey on 112 multifamily dwellings, and the principal findings showed that families with financial constraints spent more time below 18 °C, which may seriously affect their thermal comfort and health [18]. Therefore, large-scale investigation of indoor temperature of housing is important for understanding the comfort, health, and living conditions of local residents. However, it is difficult to obtain residential indoor temperature data in large-scale. There exit two practical issues conducting large-scale indoor temperature survey. First, placing temperature sensors house-by-house in region-, or even city-scale results in large instrument and labor costs. Second, direct temperature measurement requires entering each household to place sensors, which interferes with the daily lives of the residents.

Infrared thermography, a nonintrusive technique for measuring temperature, might be a breakthrough for large-scale indoor temperature investigation. As a prominent tool for detecting infrared energy emitted by object surfaces, infrared thermography has been widely applied in building diagnostics, such as determination of the overall thermal conductivity (U-value) of exterior walls [19–21] and windows [22–24] and the detection of heat loss points [25–27], air leakages [28–30], and moisture [31–33] of the building envelope. Several attempts have been made to employ infrared thermography to measure indoor temperature. Lee et al. proposed a method to measure the indoor mean radiant temperature by using a pan-tilt camera to detect the interior surface temperatures [34]. Fokaides et al. used infrared camera to measure the indoor air temperature by placing mock target material interior the test room [35,36]. They experimentally proofed that the surface temperature of the mock target can present indoor air temperature when thermal equilibrium is reached. Porras-Amores et al. developed a method of using infrared cameras to measure the distribution of indoor air temperature by setting highly reflective screens inside the room. [37].

Previous studies on indoor temperature measurement using infrared thermography were conducted interior the rooms. Practically, it is difficult and time consuming to enter the rooms and place materials for indoor temperature measurement house by house during the indoor temperature survey. In this regard, we were inspired by the question that, based on the remote sensing property of infrared thermography, is there any way to detect the indoor temperature from building outside, without the need of interrupting the occupants?

In this paper, a novel method is proposed to use infrared thermography to identify the indoor temperature remotely by opening window hole from outside the building. Different from previous studies, one could obtain indoor temperature data from building outside by applying this method. This method, if successful, could lay a foundation for obtaining room temperature data from remote view infrared images. In this manuscript, an experimental study was conducted to explore the feasibility and accuracy of this method. The experimental results were analyzed and limitations of the proposed method were discussed.

## 2 Methodology

**Fig. 1:**
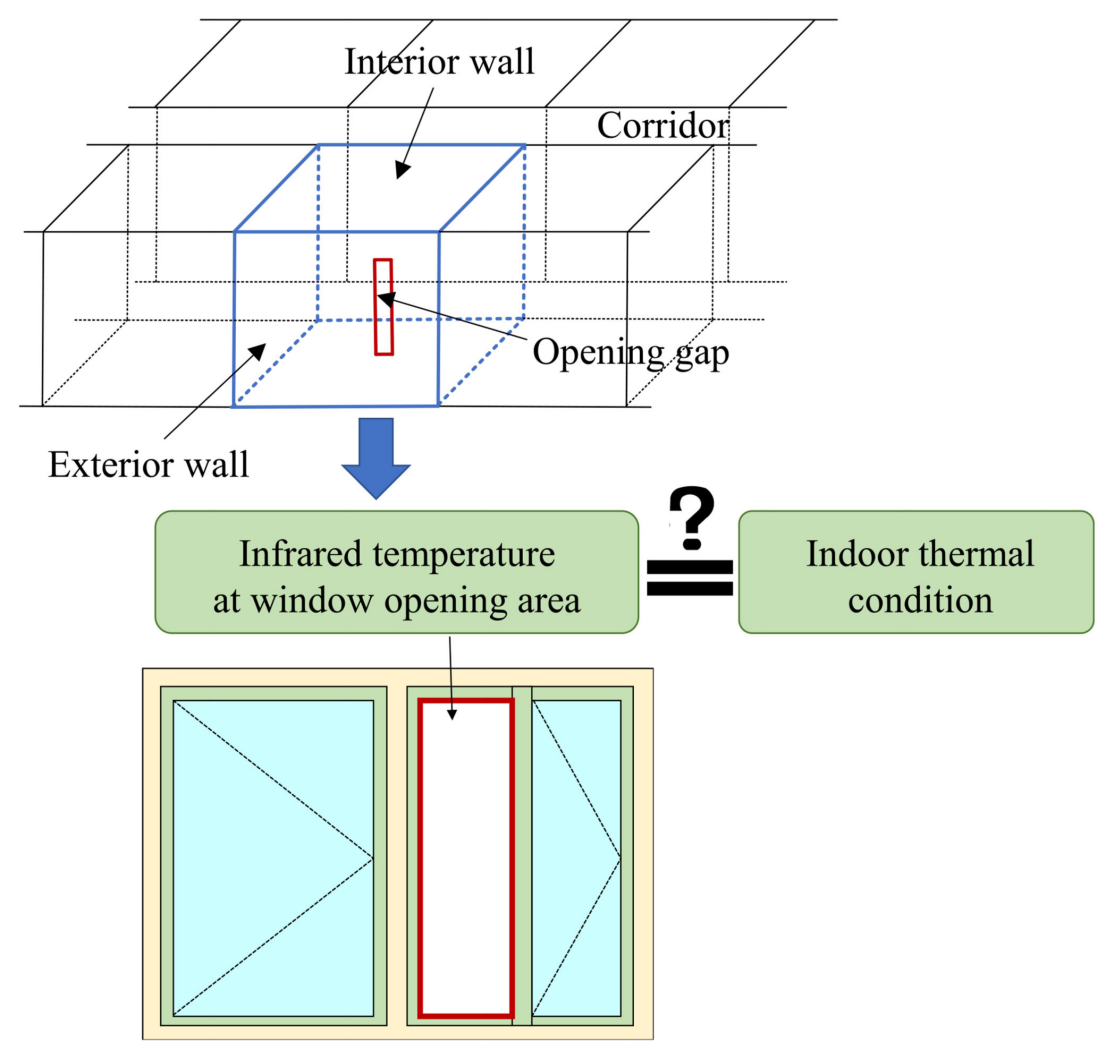
Hypothesis of detecting the indoor thermal environment through an infrared image at the window opening gap.

From the infrared images of building envelopes, it was observed that the detected temperature at the window opening gap differs from the temperature of other building envelope surface areas when windows are occasionally opened. Little attention has been paid to obtaining information about the temperature at the window opening. As shown in Fig. 1, because rooms in multistory buildings (the most common building type in city centers) are usually connected with corridors or conditioned adjacent rooms, a relatively stable indoor thermal condition can be considered in these residential buildings. This means that the room interior surface temperature is similar to the room air temperature. Therefore, the hypothesis was made that the infrared temperature detected at the window opening gap reflects the indoor thermal environment to a certain level. Based on this assumption, an experimental study was conducted to explore the following questions:

- Does the detected infrared temperature reflect the room temperature?
- To what extent can the infrared temperature reflect the room temperature?
- What influences the accuracy of this method?

To answer these questions, an experiment in a conditioned test room was conducted. Purpose of the experiment was to explore the possibility and feasibility of remotely sensing the indoor thermal environment by using infrared camera outside the building.

### 2.1 Experiment facility

**Fig. 2:**
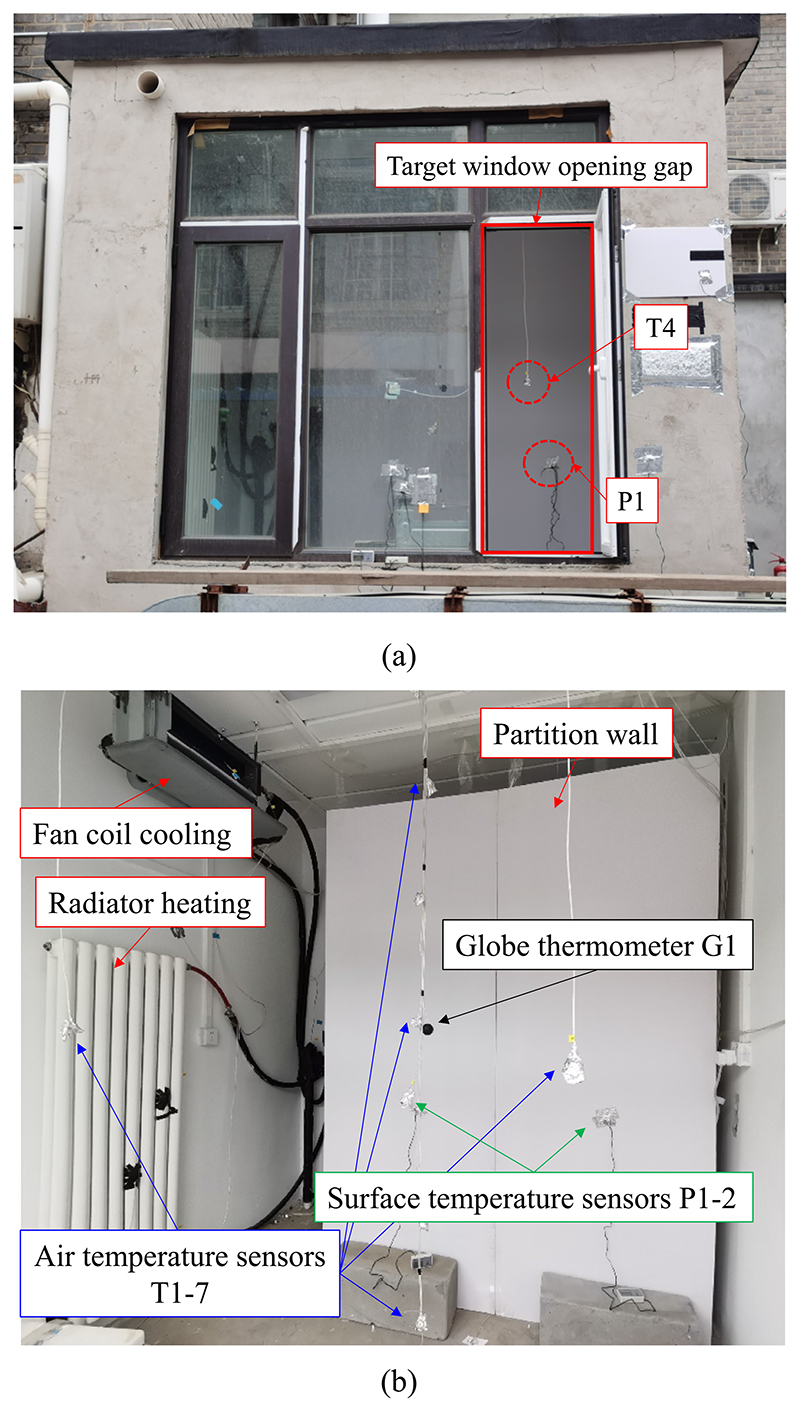
Photograph of the test room: (a) outside view and (b) inside view.

**Fig. 3:**
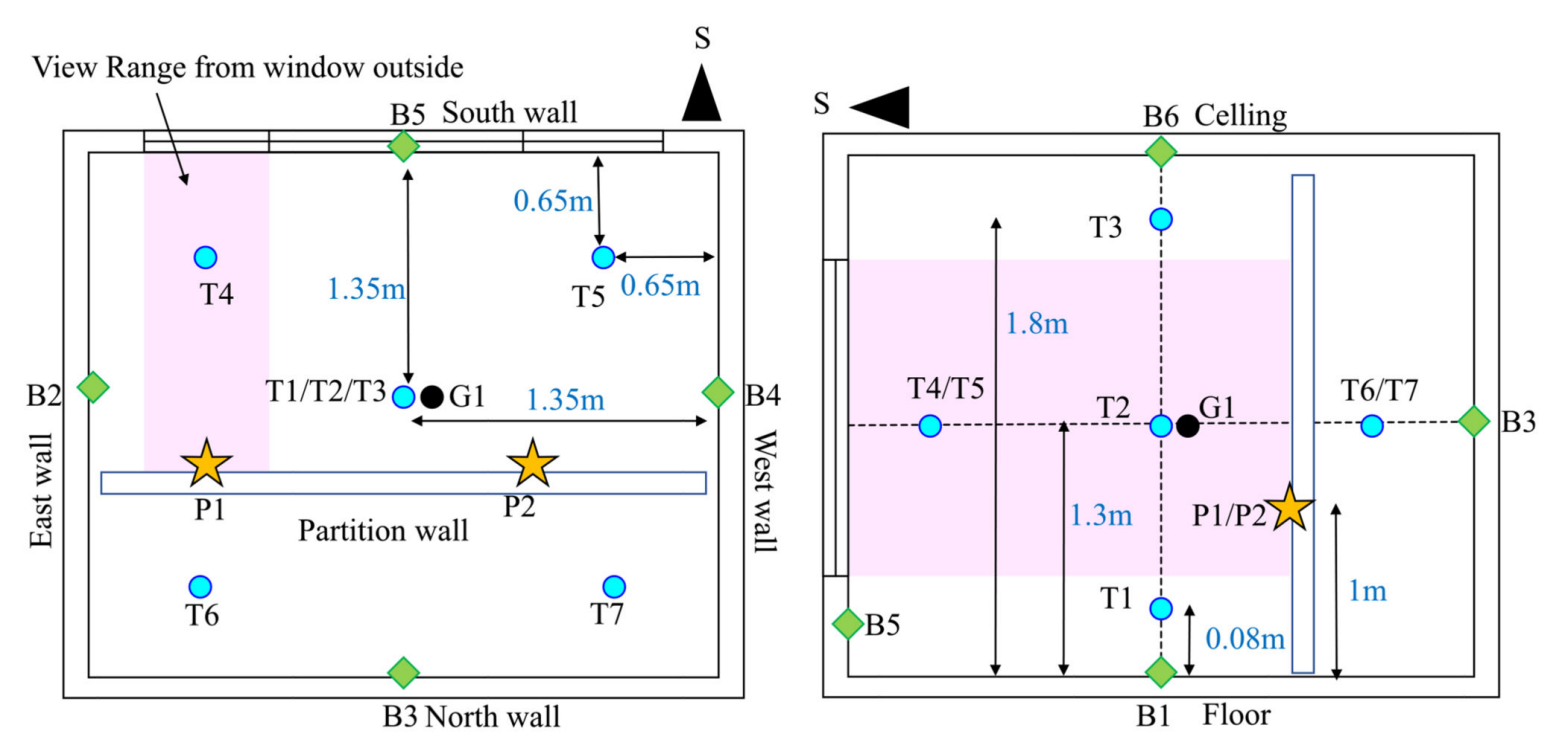
Positions of air temperature sensors T1–T7, surface temperature sensors of room interior surfaces B1–B6, and surface temperature sensors of partition walls P1 and P2.

**Fig. 4:**
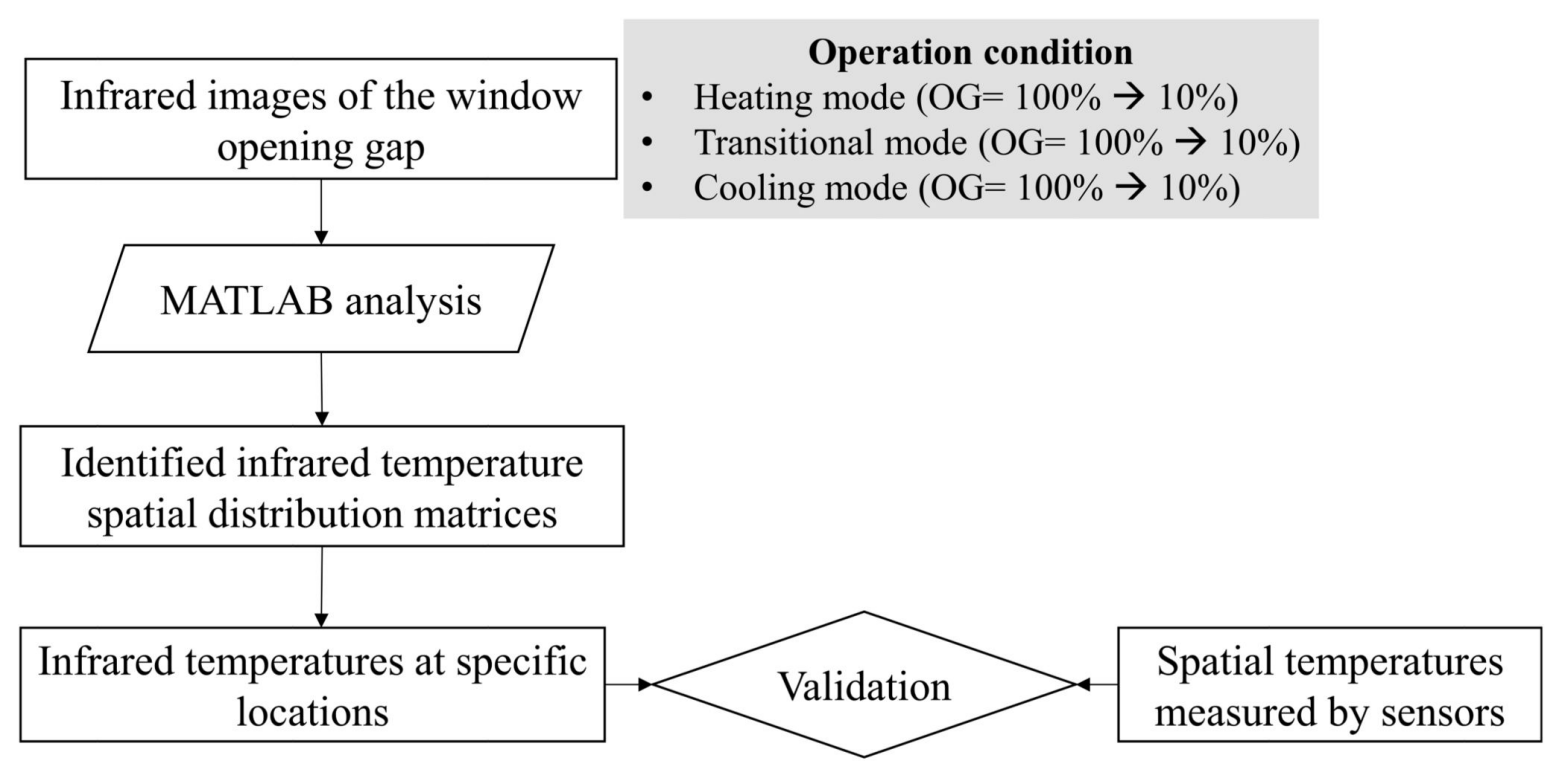
Validation progress (OG is the window opening grade).

The room used in the experiment is located on the campus of Tsinghua University in Beijing, China. This southerly oriented room has interior dimensions of 2.7 × 2.7 × 2.7 m. All the walls of the room are exterior walls made of 30-cm-thick concrete. As shown in Fig. 2(a), the room has a large exterior window on the south wall. During the experiment, the right side window remained open to form a hole through which the infrared camera could detect the interior condition. To simulate a room condition with an interior wall, a lightweight partition wall made of plastic board was built inside the room (Fig. 2 (b)). The indoor thermal condition of the test room could be controlled by fan coil cooling and radiator heating. The inlet and outlet pipes of the fan coil and radiator were connected to a thermostat water tank located in the equipment room behind the test room. Different temperature sensors were installed in the room to monitor the indoor thermal environment. As shown in Fig. 3, three air temperature sensors (T1–T3) were positioned along the room central line with heights of 0.08, 1.3, and 1.8 m, respectively. Four air temperature sensors (T4–T7) were arranged at the four corners at a height of 1.3 m. One globe thermometer was installed at the center of the room at a height of 1.3 m. The temperatures of the six interior surfaces were monitored by corresponding surface temperature sensors (B1–B6). In addition, two temperature sensors (P1 and P2) were positioned on the front surface of the partition wall at a height of 1 m. All the probes of the temperature sensors were wrapped in tinfoil to prevent the interference of short-wave solar radiation and long-wave radiation from the environment. Viewed from the horizontal direction outside the room, the measurement points that could be directly seen from the window opening were the air temperature sensor T4 and surface temperature of the partition wall P1 (see Fig. 3(a) and 4(a)). All temperature sensors were Pt 100 with an accuracy of ± 0.2 °C. The measurement data were recorded in an Agilent data logger at time intervals of 10 s.

### 2.2 Experimental setup

The experiment was conducted in June 2021. The infrared camera VarioCAM HiRes produced by Jenoptik was used. The infrared camera was placed 5 m from the target opening window. The height of the infrared camera tripod was adjusted so that camera lens maintained a horizontal shooting view to the target window. To avoid the influence of solar radiation on the infrared photography, experiments were conducted at night from approximately 19:30 to 20:30. During the experimental period, the infrared images were recorded at time intervals of approximately 60 s. To investigate the effect of the window opening size on the remote-sensing accuracy, the window opening grade gradually decreased from 100% to 10% during each filming time. The opening grade is defined as the ratio of the visible opening area from the horizontal view to the maximal opening area. To investigate the possible influence of the temperature difference between the indoor and outdoor environments on this method, the indoor thermal environment was controlled by a different operation mode during each experiment. As illustrated in Table 1, three operation modes were employed separately. When the room was in heating mode, hot water was supplied to the radiator to heat the room. In cooling mode, cold water was supplied to the fan coil under the ceiling. The fan coil sent cold air to cool the room. In the transitional mode, the cooling and heating loops were shut down. To keep the room temperature as stable as possible during the night filming period, the radiator heating or fan coil cooling was performed 10 h before the experiment started to ensure that the temperature fluctuation of each interior measuring point did not exceed 1 °C during the experimental period [38,39].

### 2.3 Validation process

The purpose of the experimental study is to validate the feasibility and accuracy of this method. Since the infrared image provides the spatial distribution information of indoor temperatures, and numbers of temperature sensors were arranged at different locations inside the test room. For the purpose of quantitative validation, the infrared temperatures at specific locations were extracted and compared with the measured temperatures of sensors at the corresponding locations. The validation process is detailed in Fig. 4, infrared images with the window opening grade (OG) ranging from 100% to 10% were imported into MATLAB. The written program reads the red–green–blue (RGB) value of each pixel in the images and generates the corresponding temperature matrices by comparing the RGB value of each pixel with the temperature color scale. The identified infrared temperatures at specific spatial locations were extracted and compared with the measured room temperatures at corresponding locations.

## 3 Results

### 3.1 Infrared temperature identification

**Fig. 5:**
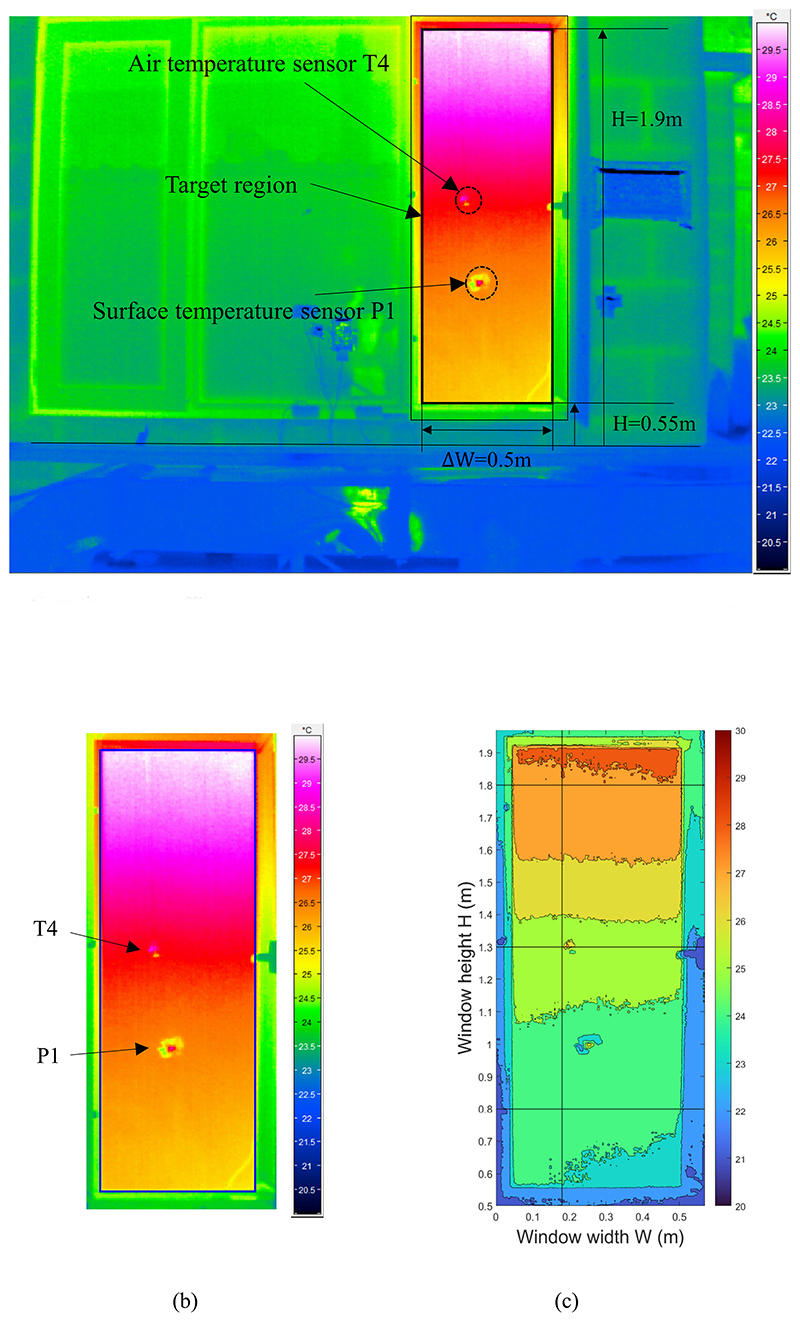
Infrared image of room in heating mode at 100% window opening grade: (a) original image, (b) target opening gap, and (c) detected temperature spatial distribution matrix.

Fig. 5(a) shows a full-size infrared image taken when the room was in heating mode with the side window fully open. The infrared temperature of the window opening hole was significantly higher than those of the other areas because the average room air temperature was approximately 5.3 °C higher than the ambient temperature during the test. Owing to the high reflectance of the tinfoil, air temperature sensor T4 (height: 1.3 m) and partition wall surface temperature P1 (height: 1.0 m) in the sight area was bright white. For the fully open window, the indoor temperature information of a rectangular area with a width of 0.5 m and height of 0.55–1.9 m could be obtained from outside the room. By converting the rectangular region into a temperature matrix using MATLAB, the spatial distribution of the detected indoor infrared temperature was obtained, as shown in Fig. 5(c). The room temperature shows a stable vertical layering under radiator heating.

**Fig. 6:**
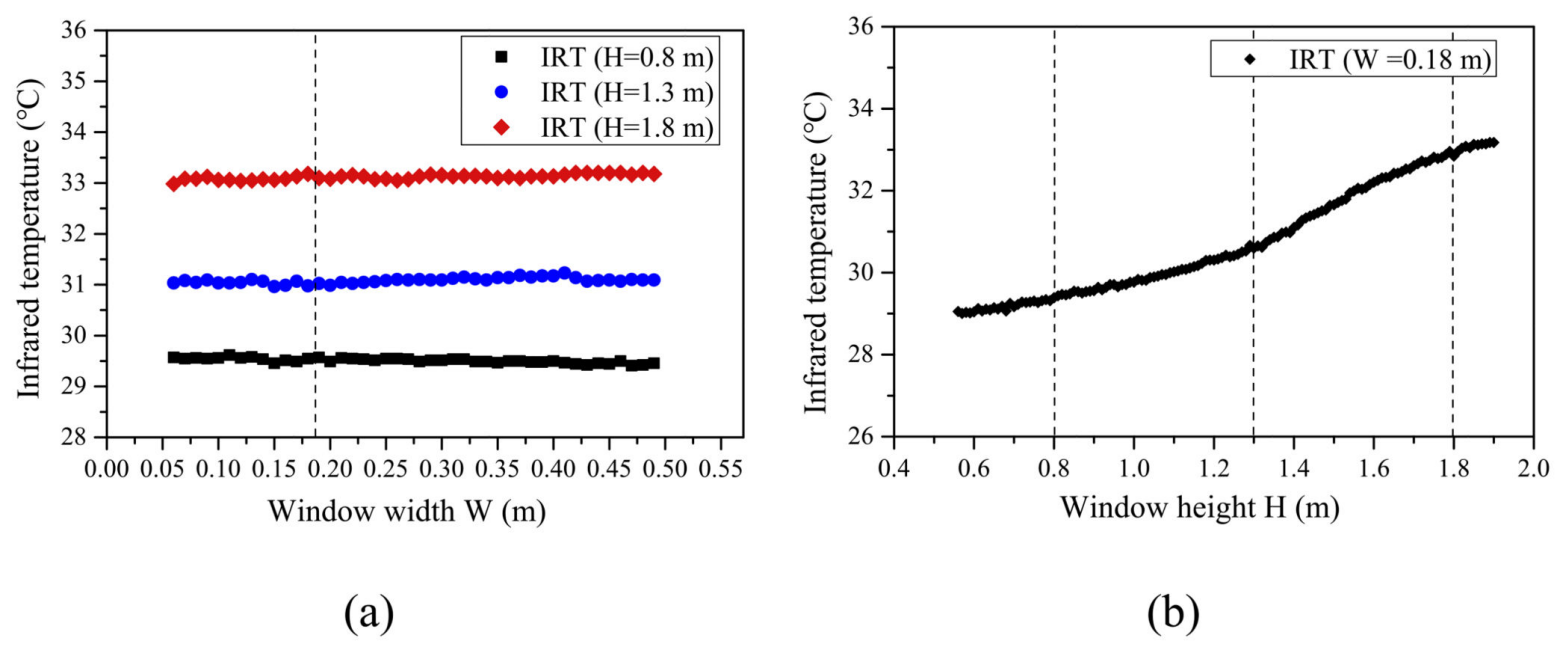
Detected infrared temperature (IRT) in heating mode at a window opening grade of 100%: (a) horizontal distribution and (b) vertical distribution.

Fig. 6(a) shows the horizontal infrared temperature distribution at heights of 0.8, 1.3, and 1.8 m, respectively. The infrared temperature distribution along the height (W = 0.18 m) is presented in Fig. 6(b). The indoor infrared temperature has an almost uniform distribution along the horizontal direction and a gradually accelerating temperature rise along the vertical direction. The infrared temperature increased 1.6 °C when the height increased from 0.8 to 1.3 m and 2.0 °C when the height increased from 1.3 to 1.8 m.

**Fig. 7:**
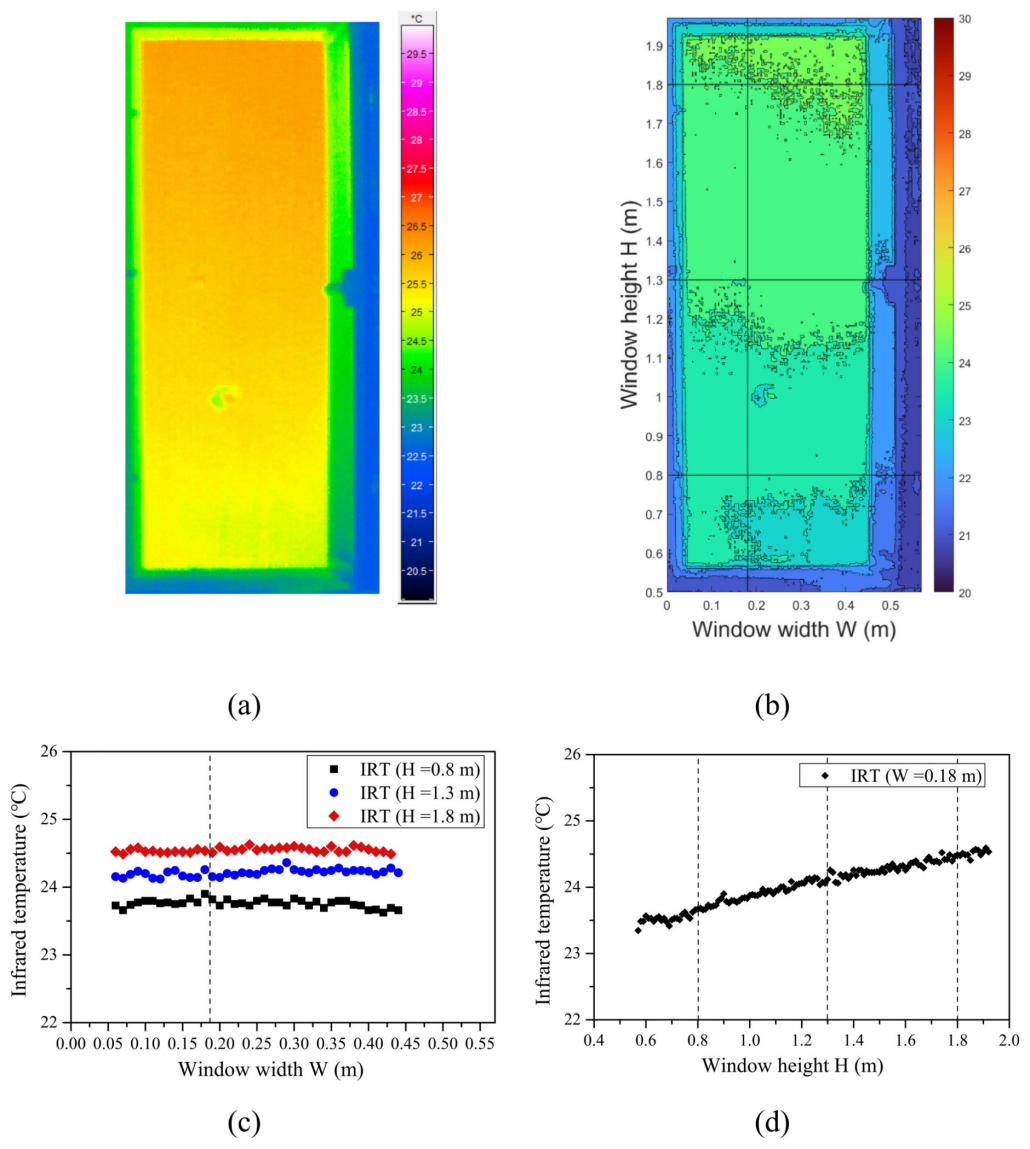
Infrared image in transitional mode at window opening grade of 100%: (a) infrared image of the window opening gap, (b) detected temperature spatial distribution matrix, (c) horizontal temperature distribution, and (d) vertical temperature distribution.

The same method was used to analyze the infrared image in the transitional and cooling modes. Fig. 7 shows the infrared temperature matrix in the transitional mode with the window fully open. As Fig. 7(b) reveals, the infrared temperature had a relatively uniform distribution in the horizontal direction under natural conditions. A slight temperature rise was observed in the vertical direction. The temperature at a height of 1.3 m was 0.5 °C higher than the one at a height of 0.8 m and 0.3 °C lower than the one at height of 1.8 m.

**Fig. 8:**
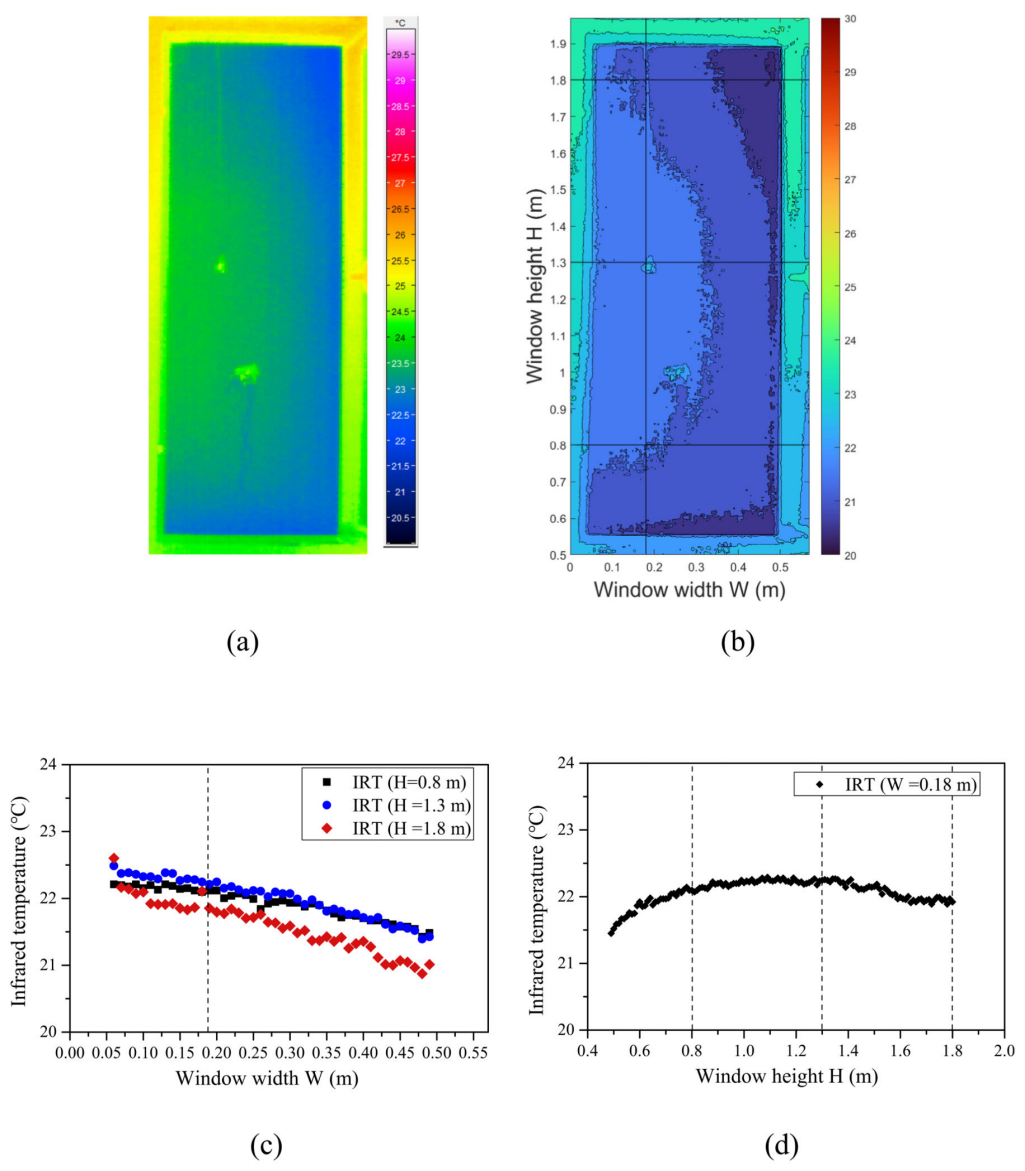
Infrared image in room cooling mode at window opening grade of 100%: (a) infrared image of the window opening gap, (b) detected temperature spatial distribution matrix, (c) horizontal temperature distribution, and (d) vertical temperature distribution.

Fig. 8 illustrates the detected infrared temperature matrix in cooling mode with the window fully open. The infrared temperature distribution is in a circulation shape — warmer near the room center and cooler near the edge. The infrared temperature distribution trend is consistent with the jet flow formed by the cold air supplied by the ceiling fan coil. Influenced by the cold jet flow, the infrared temperature distribution is non-uniform from the vertical and horizontal directions (see Fig. 8 (c) and (d)).

Analyzing three operation conditions (radiator heating, natural condition, and fan coil cooling) reveals that the infrared temperature data at the window opening gap provide the spatial distribution information of the room temperature to a certain extent. To investigate the degree of accuracy with which the detected infrared temperature reflects the indoor thermal environment, the infrared temperature distribution was compared with the measured temperature data in the next section.

### 3.2 Comparison between infrared temperature and measured data

#### 3.2.1 Heating mode

**Fig. 9:**
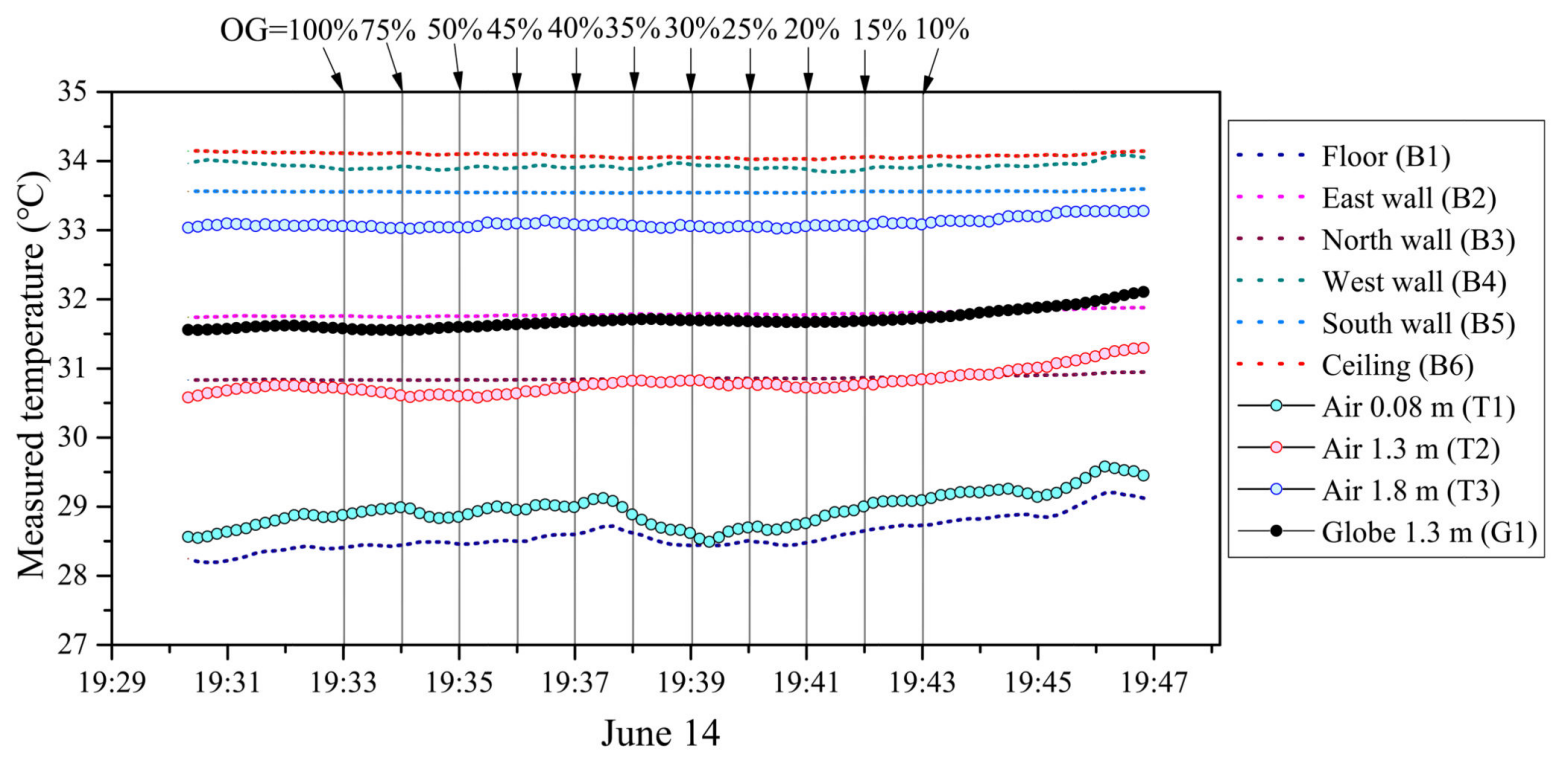
Measured data of indoor air temperature, globe temperature, and surface temperature of interior room surfaces in heating mode (June 14, 2021): OG is the window opening grade (100%–10%).

Fig. 9 shows the temperature sensor data of different locations during the camera-screening period when room was in heating mode. In order to explore the effect of window opening grade (OG) on the identify accuracy, the window opening was gradually transiting from fully opening (OG = 100%) to almost closing (OG = 10%) during the experiment, and the infrared images at certain opening levels were filmed. The vertical dotted lines indicate the time scales for taking photos with different window opening grades. The status changes of window opening were controlled manually and kept in interval around 1 min. The surface temperature points are shown by dotted lines of different colors, the air temperature points are shown by bubbles of different colors, and the globe temperature at the center of the room is represented by black bubbles. The experiment was completed in 10 min by taking infrared images with a decreasing window opening grade at 1-min intervals. During the test, the indoor thermal environment was relatively stable — the fluctuation of the measured temperature did not exceed 0.5 °C. The highest temperature was at the ceiling surface (red dotted line) with a value of approximately 34 °C, owing to the double effect of direct solar radiation and natural convection flow driven by radiator heating. The next-highest were the surface temperatures of the west wall (green dotted line) and south wall (blue dotted line). The floor had the lowest surface temperature (~28.5 °C). Influenced by the interior surface temperature difference and natural buoyancy flow, the indoor air points along the room central line (at 0.08, 1.3, and 1.8 m) showed a stable vertical temperature gradient. The globe temperature (1.3 m) was approximately 1 °C higher than the air temperature at a nearby point. The reason could be that the globe temperature was affected by the warm wall and ceiling surfaces.

**Fig. 10:**
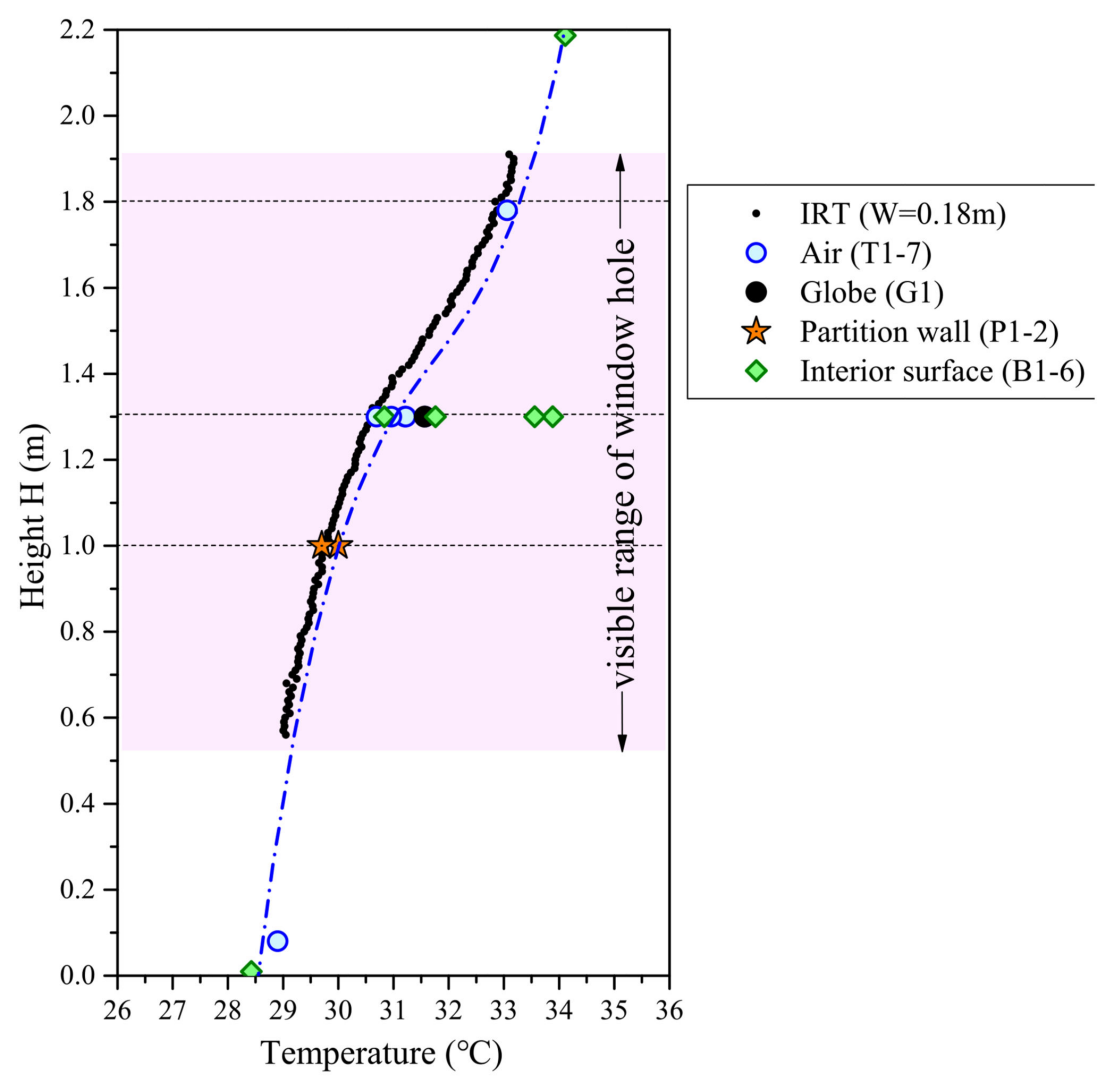
Comparison between the vertical distribution of the measured temperature data and vertical distribution of the infrared temperature (IRT) at a window width of 0.18 m in heating mode at a window opening grade of 100% (June 14, 2021, 19:33).

The measured temperature points distributed by height at the camera capture moment of a window opening grade of 100% (19:33) are shown in Fig. 10. The temperature points of the room interior surfaces are in green rhombi, air temperature points are in blue bubbles, surface temperature points of the partition wall are in orange pentagrams, and globe temperature is in a black bubble. The air and surface temperatures show a consistent monotonic temperature rise in the vertical spatial distribution, excluding the surface temperatures of the west and east walls (1.3 m), which are strongly affected by solar radiation. By fitting the values of the measured points at different heights, i.e., air temperature (T1–T7), partition wall surface temperature (P1 and P2), and surface temperature of floor and ceiling (B1 and B6), the trend line shown in the figure (blue dotted line) was obtained. For comparison, the vertical distribution of the infrared temperature is also plotted in Fig. 10 in small black points (W = 0.18 m, i.e., the column of values near measuring points T4 and P1). The vertical spatial distribution of the infrared temperature matches the measured room temperature well. At a height of 1.0 m, the average difference between the infrared temperature and the two surface temperatures of the partition wall was 0.2 °C. At a height of 1.3 m, the average difference between the infrared temperature and five air temperatures was 0.5 °C. At a height of 1.8 m, the difference between the infrared and air temperatures was 0.2 °C.

#### 3.2.2 Transitional mode

**Fig. 11:**
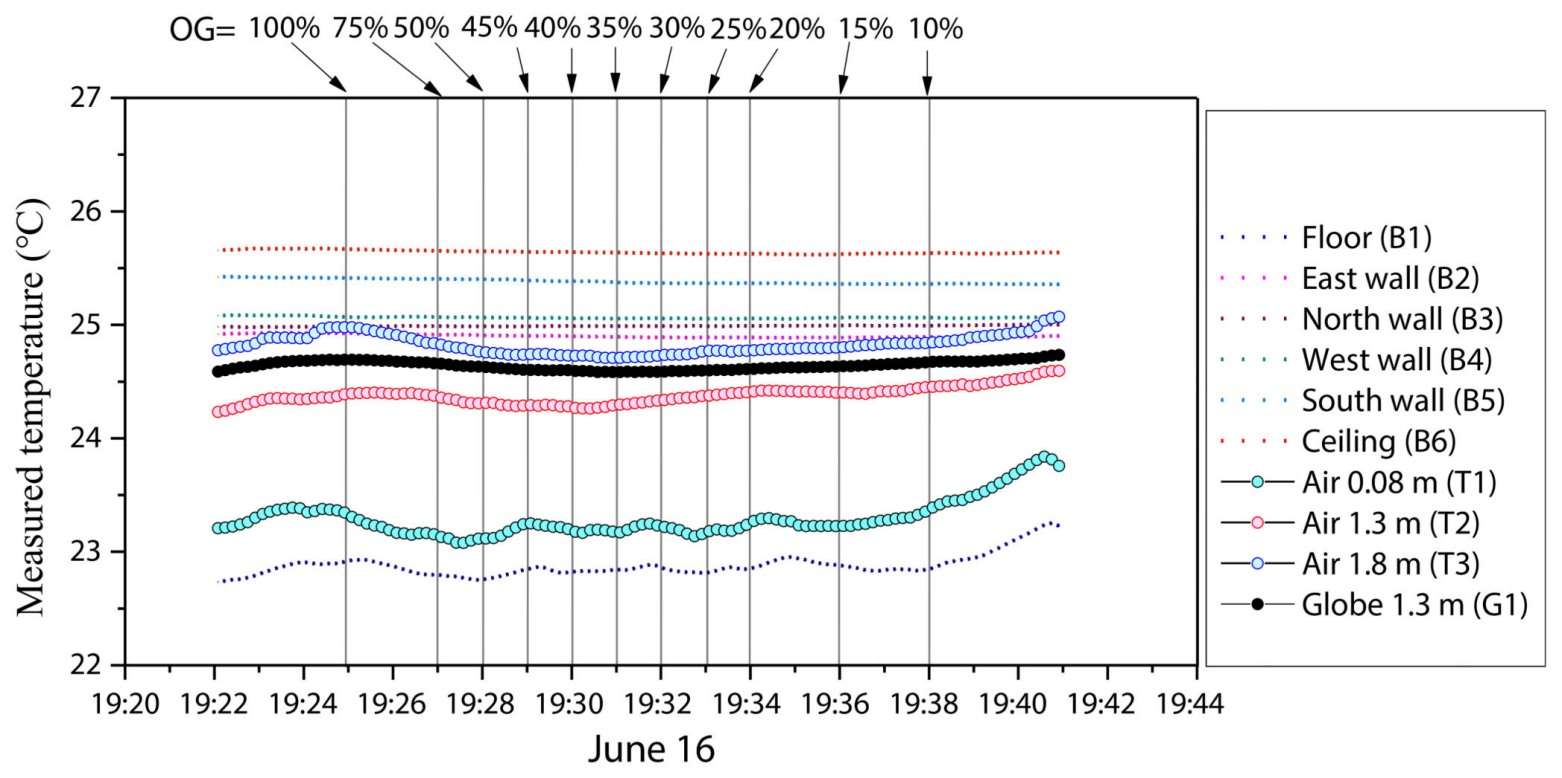
Measured data of indoor air temperature, globe temperature, and surface temperature of interior room surfaces in transitional mode (June 16, 2021): OG is the window opening grade (100%–10%).

Fig. 11 shows the measured room temperature when the room was under natural conditions without heating air conditioning devices operation. A slight vertical temperature gradient existed under natural conditions. During the camera filming period, the average air temperature difference between the points at 0.08 m (green bubbles) and 1.3 m (red bubbles) was 1.1 °C, and the difference between the points at 1.3 m (red bubbles) and 1.8 m (blue bubbles) was 0.5 °C. The interior surface temperature difference between the ceiling (red dotted line) and floor (navy dotted line) was approximately 2.8 °C. The globe temperature on average was 0.3 °C higher than the nearby air temperature, influenced by the warm ceiling and wall surfaces.

**Fig. 12:**
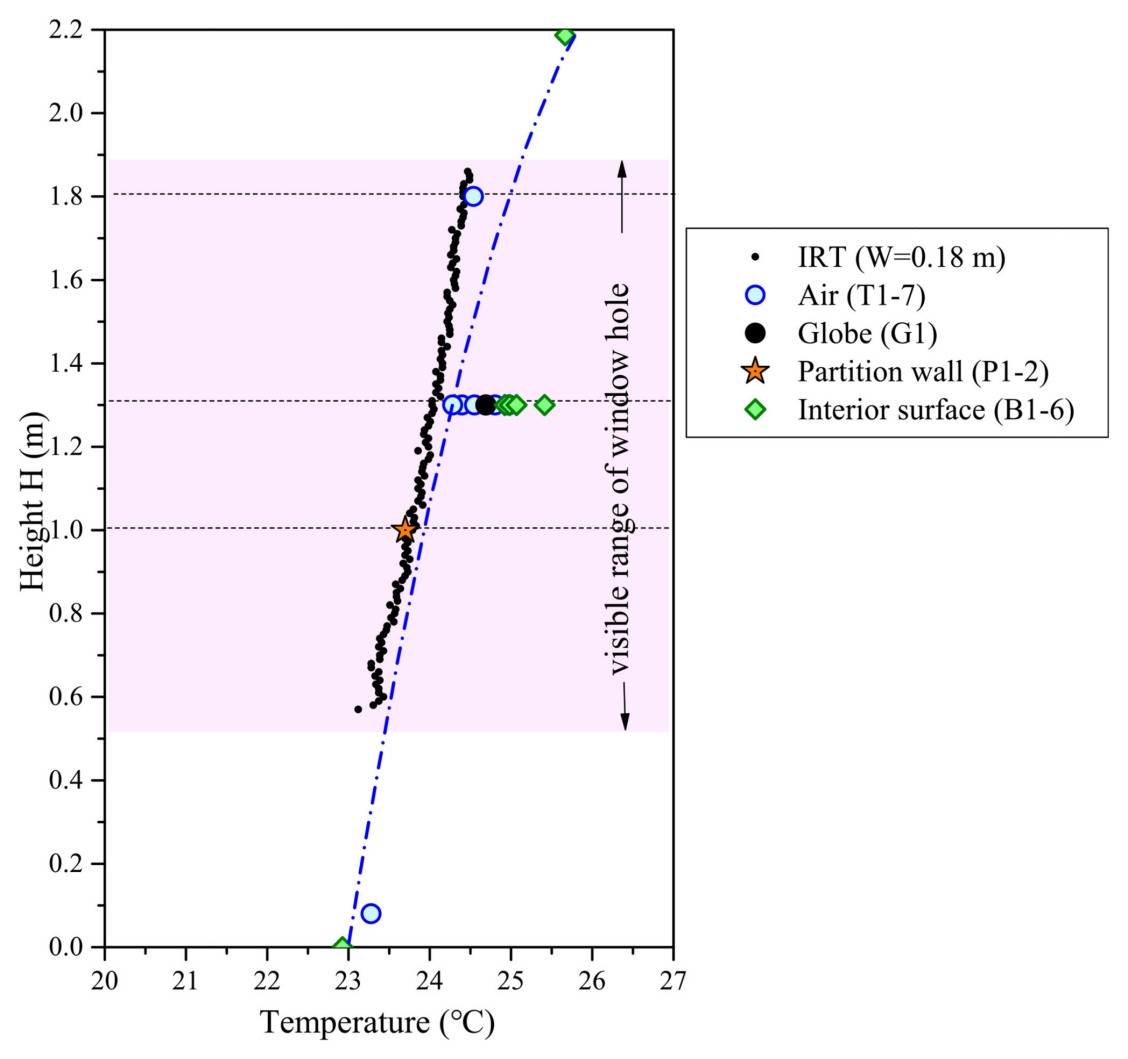
Comparison of the vertical distributions of measured temperature data and infrared temperature (IRT) at window width of 0.18 m in transitional mode at window opening grade of 100% (June 16, 19:25).

The spatial distribution of the measured temperatures at 19:25 (window opening grade of 100%) in the transitional mode is plotted in Fig. 12. The blue dotted line represents the vertical distribution trend of the air temperature (T1–T7), partition wall surface temperature (P1–P2), and surface temperature of floor and ceiling (B1 and B6). In the lower height range, the vertical distribution of the detected infrared temperatures is consistent with the trend line of the measured room temperatures, and the trend line slightly deviates when the height exceeds 1.3 m. The deviation in a higher height range is caused by the warm ceiling, which is heated by solar radiation in daytime. However, in the visible height range of the window opening gap, the infrared temperature agrees well with the measured air and surface temperatures of the partition wall. At a height of 1.0 m, the difference between the infrared and average partition wall surface temperatures is 0.2 °C. At a height of 1.3 m, the difference between infrared and average air temperatures is 0.5 °C. At a height of 1.8 m, the difference between infrared and air temperatures is 0.2 °C.

#### 3.2.3 Cooling mode

**Fig. 13:**
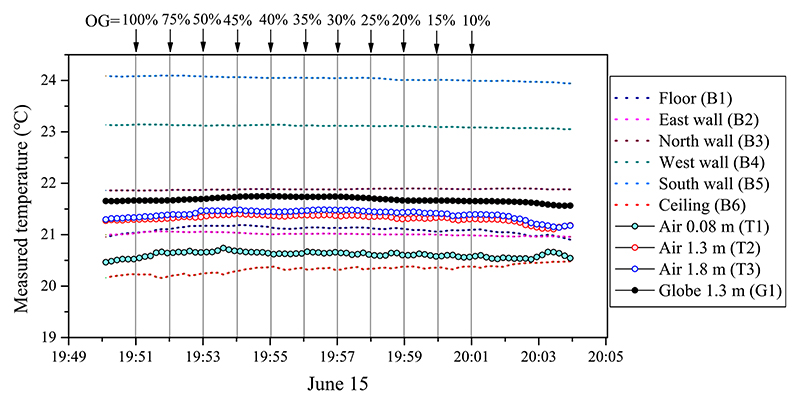
Measured data of indoor air temperature, globe temperature, and surface temperature of interior room surfaces in transitional mode (June 15, 2021): OG is the window opening grade (100%–10%).

Fig. 13 shows the transient data of the measured room temperature during the cooling period. When the room was in cooling mode, the fan coil positioned beneath the ceiling provided a cold jet flow to cool the room. Therefore, the ceiling had the coldest temperature compared with the other interior surfaces. Relatively high surface temperatures were detected at the south and west walls owing to solar radiation heating. Because the indoor air was well mixed by the circulating jet flow, the air temperatures at the various heights had similar and stable values within the range of 20.5 °C–21.5 °C.

**Fig. 14:**
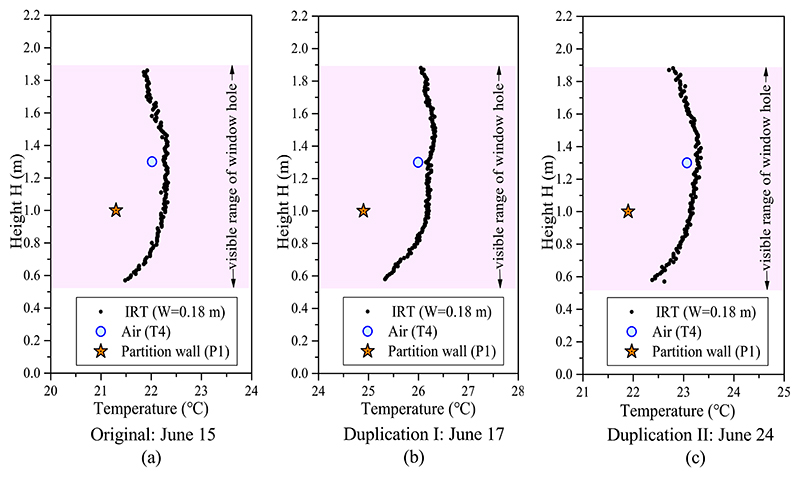
Comparison of the vertical distributions of measured temperature data and infrared temperature (IRT) at window width of 0.18 m in transitional mode at window opening grade of 100%: (a) experiment on June 15, (b) replication experiment I on June 17, and (c) replication experiment II on June 24.

As discussed in Section 3.1, unlike the heating and transitional modes in which the room temperature showed a relatively stable vertical gradient, the indoor temperature in cooling mode had a circulating form driven by the cold jet flow. Therefore, the room temperature in cooling mode was asymmetrically distributed in the horizontal and vertical directions. In this situation, the T4 air temperature and P1 partition wall surface temperature (points directly seen in infrared image) and the nearby infrared temperature were plotted for comparison. As Fig. 14(a) shows, at a height of 1.0 m, the infrared temperature nearby was 0.9 °C higher than the measured P1 surface temperature. At a height of 1.3 m, the infrared temperature approaches the air temperature with a temperature difference of 0.2 °C. Unlike the heating and transitional modes, where the detected infrared temperature at the window opening gap matched the indoor room temperature well, in the cooling condition, the infrared temperature considerably overestimated the indoor thermal condition. Two replication experiments were conducted on June 17 and June 24 to verify the results. The corresponding data are plotted in Fig. 14(b) and (c). The results of the original experiment and two duplication experiments are similar. In the duplication experiments, the infrared temperature was approximately 1.2–1.3 °C higher than the measured surface temperature at 1.0 m and 0.2 °C higher than the measured air temperature at 1.3 m. A supposed reason is that the infrared camera had a higher measurement error when used to detect a surface colder than the environment in which the camera was located. A detailed explanation can be found in Section 4.2.

**Fig. 15:**
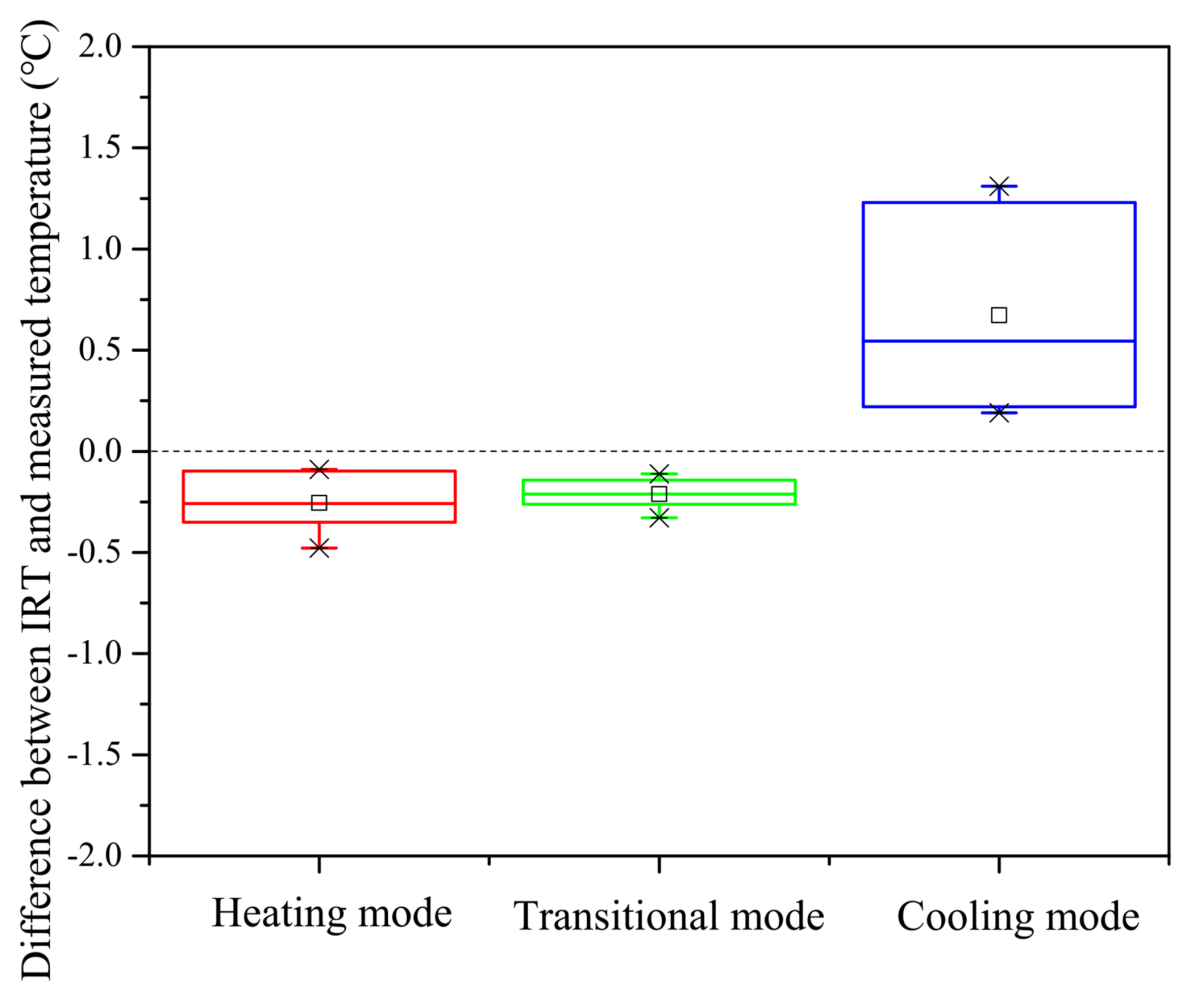
Absolute measurement errors between infrared temperature and measured room temperatures at the window opening grade of 100% under three operation conditions.

The absolute measurement errors between infrared temperature and measured room temperatures under three operation modes are summarized in Fig. 15. As shown, the identified infrared temperatures were relatively lower than the measured room temperatures at target positions under heating and transitional modes. The measurement errors under heating and transitional modes ranged from −0.5 °C to −0.1 °C and from −0.3 °C to −0.1 °C, respectively. When the room was under cooling mode, the infrared temperatures were higher than the measured room temperature, the absolute measurement error ranged from 0.2 °C to 1.3 °C.

### 3.3 Effect of the window opening grade

In practical application, occupants do not often open the windows fully. Therefore, it is necessary to explore the effect of the window opening grade on the accuracy of this method. Considering this, the window opening grade was gradually decreased in each HVAC operation mode, and the infrared images of various window opening size were analyzed. The detected infrared images with decreasing window opening size in the heating, transitional, and cooling modes are presented in Tables 2–4 respectively.

**Fig. 16:**
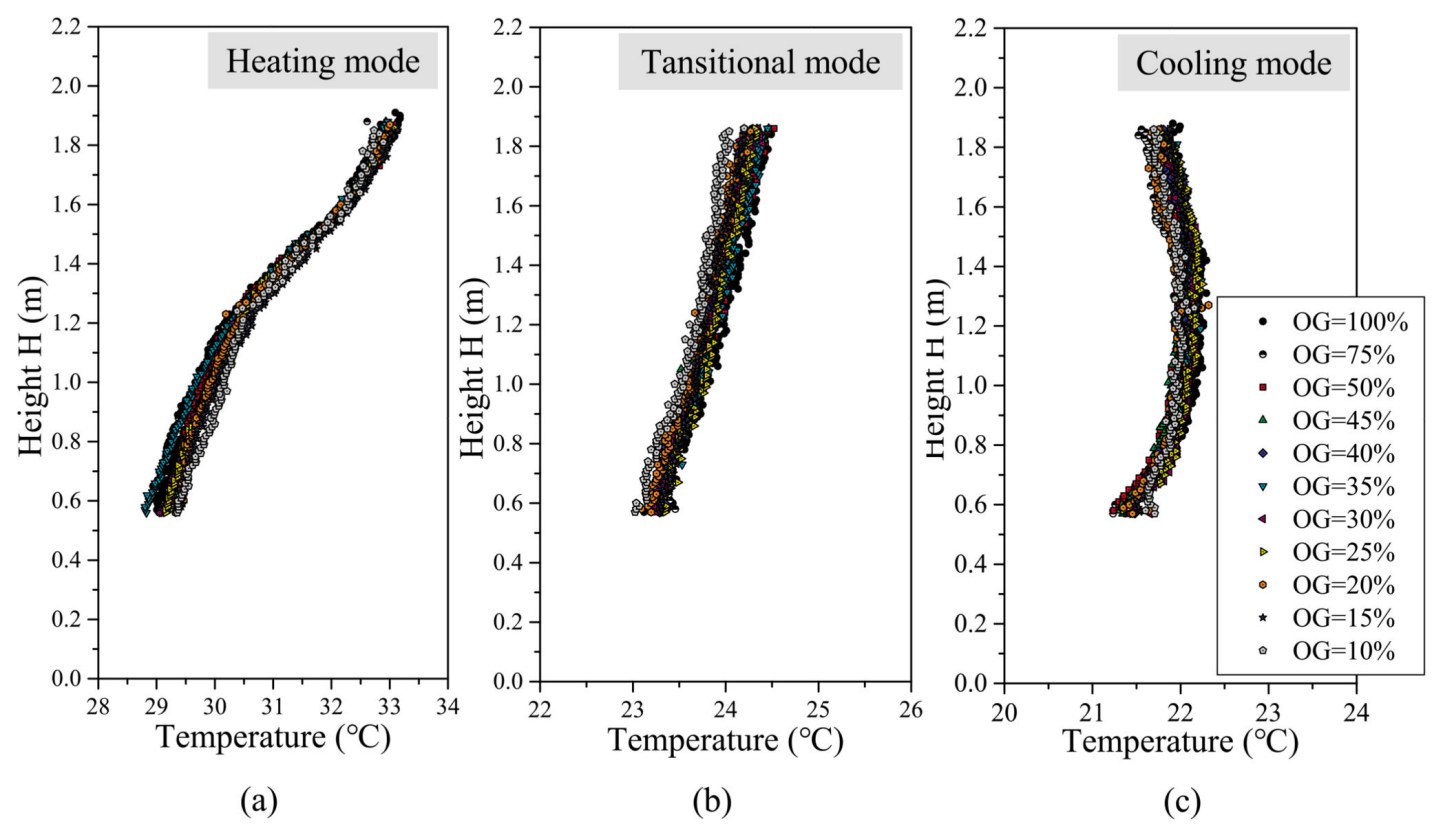
Comparison of infrared temperature at window width of 0.06 m with the window opening grade (OG) ranging from 100% to 10%: (a) heating mode, (b) transitional mode, and (c) cooling mode.

As the window opening grade decreased, less information was obtained from the infrared image at the window opening gap. When the window opening grade decreased to 10%, only a column of temperature information near the window frame was visible in the infrared image. Because there was less information acquisition, to analyze the effect of the window opening on the infrared recognition accuracy, the vertical distribution of infrared temperature near the window frame (0.06 m) from images with various window opening grades was analyzed. As plotted in Fig. 16, the sets of identified infrared temperature data at W = 0.06 m are almost identical. The deviations of the temperature points are within 0.5 °C. Since the room temperature remained relatively stable during the quick tests (less than 15 min), it can be concluded that the window opening grade did not have much influence on the accuracy of infrared temperature identification.

## 4 Discussion

### 4.1 Relationship of three temperatures

**Fig. 17:**
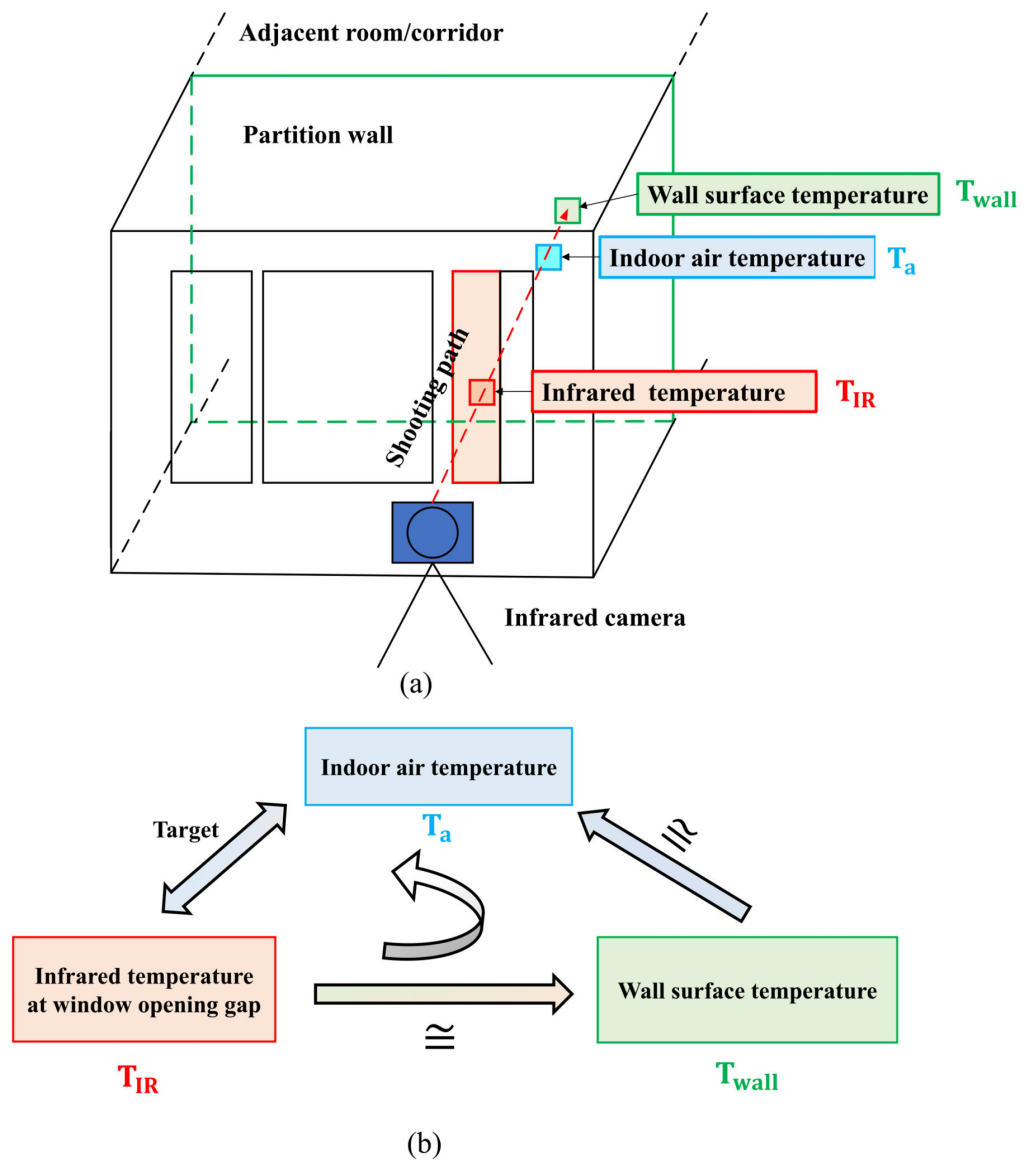
Relationship between infrared temperature at window opening gap, wall surface temperature, and indoor air temperature.

The detected infrared temperature at the window opening gap reflects the spatial distribution of the air and partition wall surface temperatures to certain extent when the room is under a relatively stable thermal condition. The physical principle of the remote measurement is shown in Fig. 17. The working principle of the infrared camera is to receive the infrared rays emitted by the object surface and obtain an image by photoelectric conversion. Therefore, the infrared temperature detected through the window opening gap indicates the surface temperature of partition wall. Assuming that the effects of heat conduction and heat capacity of the partition wall can be ignored, the surface temperature of the partition wall is a weighted average of the air temperature adjacent to the partition and radiant temperature from the interior surrounding wall surface. When the room is in a relatively stable thermal condition and the target wall connected to adjacent room or corridor has a similar temperature, the surface temperature of the partition wall, the adjacent air temperature and the radiant temperature of interior surrounding are close to each other through convective and radiant heat exchange. Therefore, as Fig. 17 (b) shows, analyzing the relationship between infrared temperature, surface temperature of the partition wall and the indoor air temperature reveals that the infrared temperature at the window opening gap reflects the spatial distribution of the indoor air temperature when certain conditions are met. In practical applications, for conventional multistory houses, the surface temperature of the interior partition wall is usually not much different from the indoor air temperature. Therefore, it is feasible to detect the indoor air temperature through infrared remote sensing from the window opening gap.

### 4.2 Influence of HVAC operation condition and window opening grade

As illustrated in Section 3.2, the infrared sensing has a relatively high accuracy when the room is under radiator heating or a natural condition. Infrared image at the window opening gap can obtain the vertical air temperature gradient with an absolute error almost within 0.5 °C. However, when the room is under fan coil cooling, although the infrared image at the window opening gap can obtain the cold circulation flow blown by the ceiling fan coil, the infrared temperature significantly overestimates the room temperature (exceeding 1 °C). Considering the working principle of the infrared camera, the supposed reason for the relatively high error for a cold surface is that, when the surface of the object is colder than the surroundings, the emitted radiant intensity is lower than that of the surroundings according to Planck’s law. The resulting disturbance might cause overestimation of the object surface temperature. Maley et al. also reported an overestimation of skin temperature by infrared sensing when the skin was colder than the surroundings after immersion in cold water [40].

In the practical applications, the window can be partially or fully opened depending on user behavior. When the window opening area is smaller, less information about the indoor thermal environment can be obtained by infrared sensing. The analysis in Section 4.3 verified that the accuracy of infrared detection remained almost unchanged with decreasing opening area. The practical significance of this finding is that an infrared camera can remotely sense the room temperature, even if the window is only slightly open.

### 4.3 Limitations and future studies

This study was conducted under laboratory conditions with a well-controlled indoor thermal environment. Following factors should be concerned in the future study.

(1) The thermal condition of the test room was relatively stable when the window was open. This might differ from the thermal condition in a real room.
(2) The experiment was conducted in an empty test room without heat sources such as occupants and electric devices, which have influence on the surrounding air temperature.
(3) The partition wall in the test room was made of lightweight plastic board. The surface temperature of the partition wall was highly consistent with the ambient air temperature. The interior wall and thermal environment of a real building might or might not be similar with the experimental condition.
(4) The experiment was conducted with an infrared camera fixed with a horizontal view at a constant distance of 5.2 m. The influence of the camera distance and angle should also be investigated in the future.

Overall, this experimental study preliminarily validated the feasibility and accuracy of the method by using an infrared camera to detect the indoor thermal environment through the window opening gap. In the further study, this method should be further validated in real buildings considering such practical issues as the thermal stability of a real building (especially with opening windows), influence of interior heat sources, camera distance and angle, sight interference caused by trees and window nets, etc.

## 5 Conclusions

This study proposed a novel method to measure indoor temperatures remotely from outside the building by using infrared camera. Compared with the traditional method by placing temperature sensors house by house, this remote sensing method could directly measure indoor temperature without the need to enter the room. Moreover, it could even possibly reflect the spatial distribution of indoor temperatures that is impossible to obtain by point measurements. The feasibility of this assumption was verified through an experiment conducted in a cement room under radiator heating, transitional, and fan coil cooling conditions. The results showed that the remote sensing of the indoor air temperature by an infrared camera is feasible when the indoor thermal environment is relatively stable. The main conclusions of this experimental study are as follows.

1) The temperature matrix identified by an infrared image taken at a fully opened window can quantitatively describe the vertical temperature gradient of the interior room environment when the room is under radiator heating or a natural condition, as well as the circulating cold air flow pattern resulting from fan coil cooling.
2) The vertical distribution of infrared temperature was highly consistent with the distribution tendency of the measured air and surface temperatures at different heights in heating and transitional mode. The absolute deviations between the identified infrared temperature and the measured room temperatures at different heights were within 0.5 °C. While in cooling mode, higher deviations were observed between the infrared and partition wall surface temperatures, with values ranging from 0.9 to 1.3 °C at 1.0 m during the original and two duplication experiments.
3) Less information about the indoor thermal environment can be obtained with a decreased window opening grade. However, the accuracy of infrared recognition does not change in the identifiable opening area.
4) These conclusions were obtained in a laboratory study with a well-controlled and stable thermal condition and light-weight partition wall. The feasibility and accuracy of this remote-sensing method in real buildings should be investigated further.

## Figures and Tables

**Table 1:** Experimental conditions of three operation modes.

	Heating mode	Transitional mode	Cooling mode
Date	6/14/2021	6/16/2021	6/15/2021
Camera filming time	19:30–19:45	19:25–19:37	19:51–20:02
Filming interval	60 s	60 s	60 s
Ambient temperature	25.7 °C	22.00 °C	25.1 °C
Globe temperature	31.7 °C	24.6 °C	21.7 °C
Mean temperature of interior surfaces	32.2 °C	24.9 °C	22.1 °C
Mean air temperature at 0.08 m	29.0 °C	23.3 °C	20.6 °C
Mean air temperature at 1.3 m	31.3 °C	24.4 °C	21.3 °C
Mean air temperature at 1.8 m	33.1 °C	24.8 °C	21.4 °C

Note: All temperatures in this table are average values during the experimental period.

**Table 2:** Infrared images in heating mode with the window opening grade ranging from 75% to 10%.

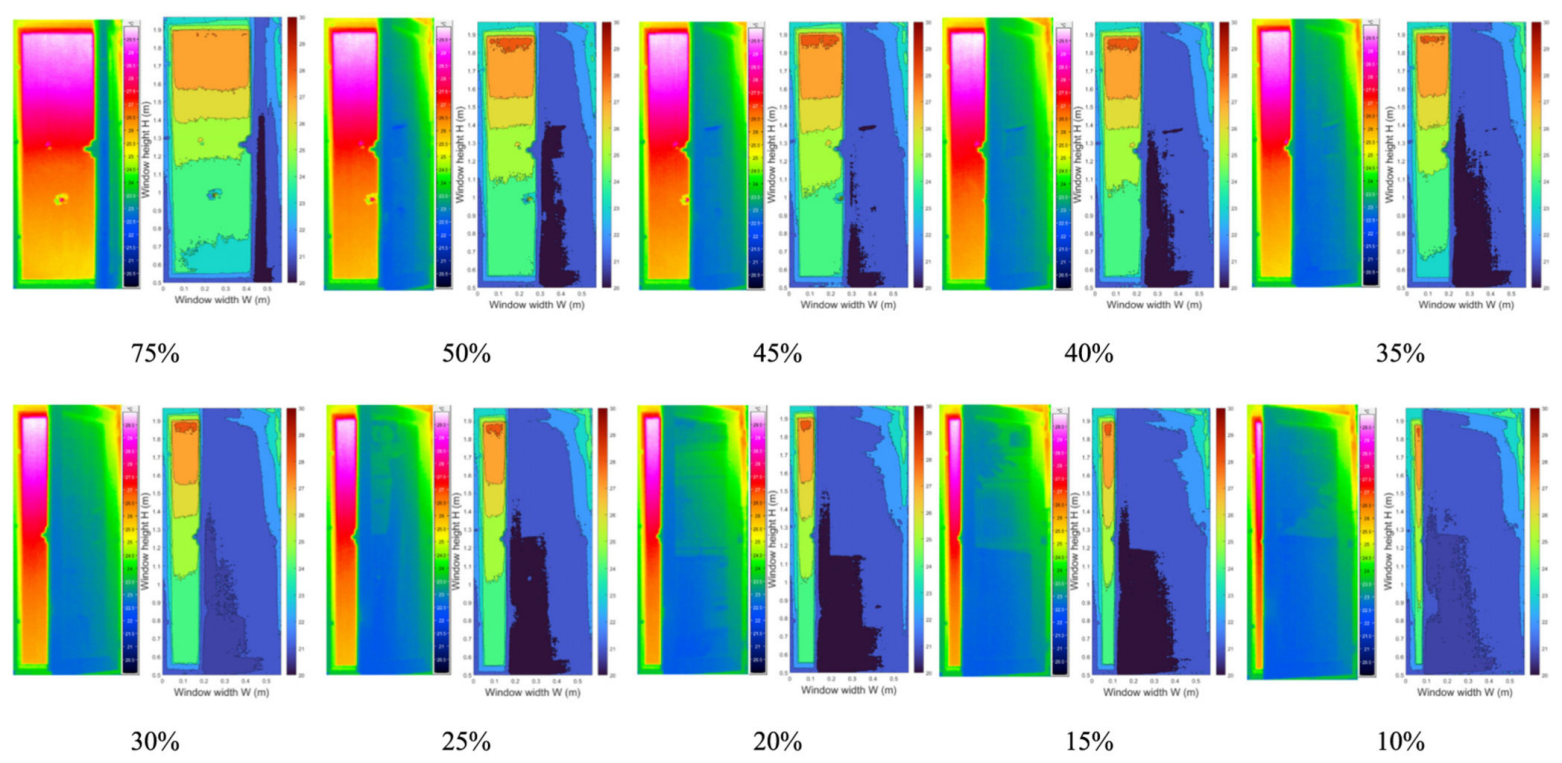

**Table 3:** Infrared images in transitional mode with the window opening grade ranging from 75% to 10%.

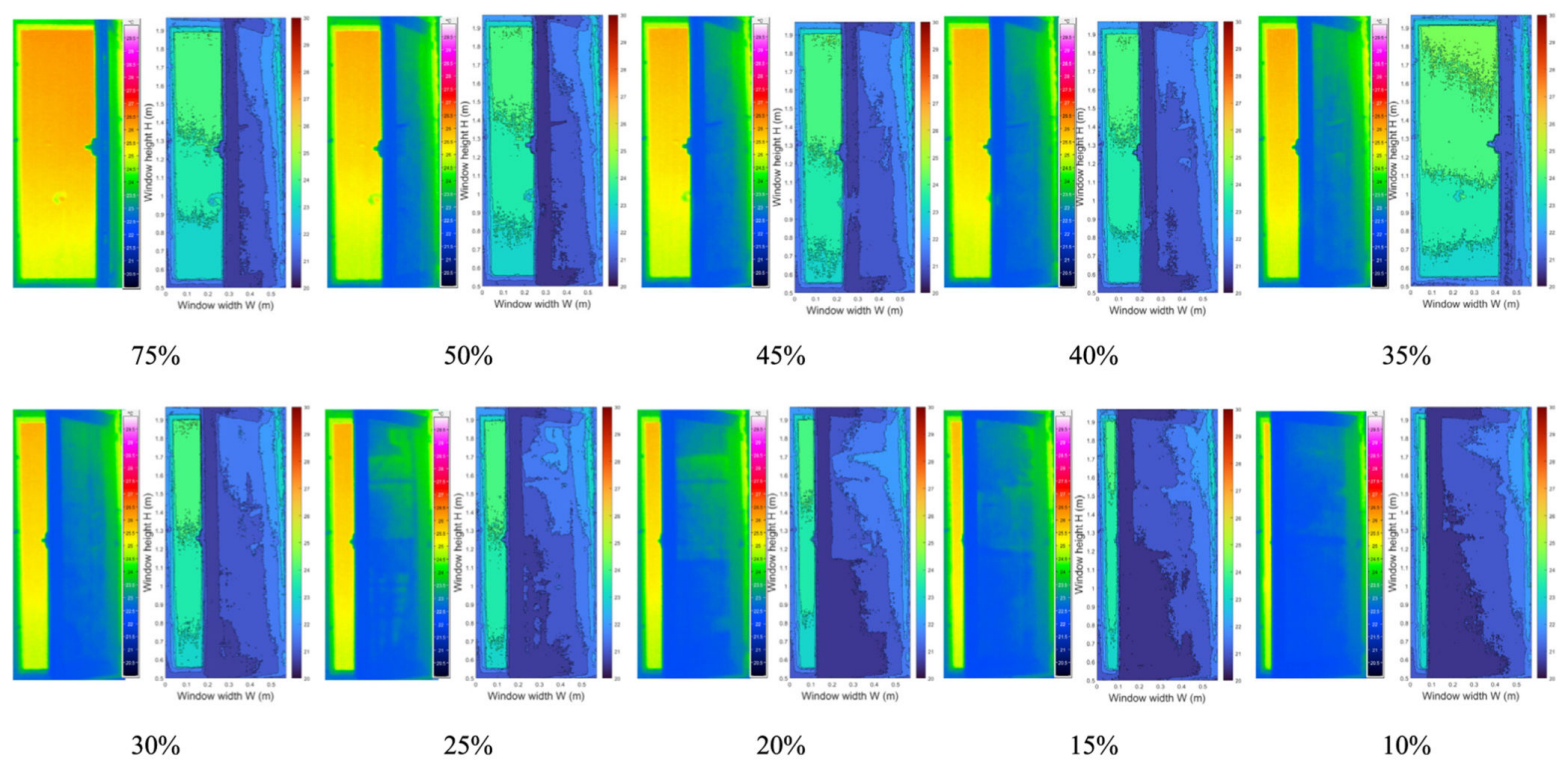

**Table 4:** Infrared images in cooling mode with the window opening grade ranging from 75% to 10%.

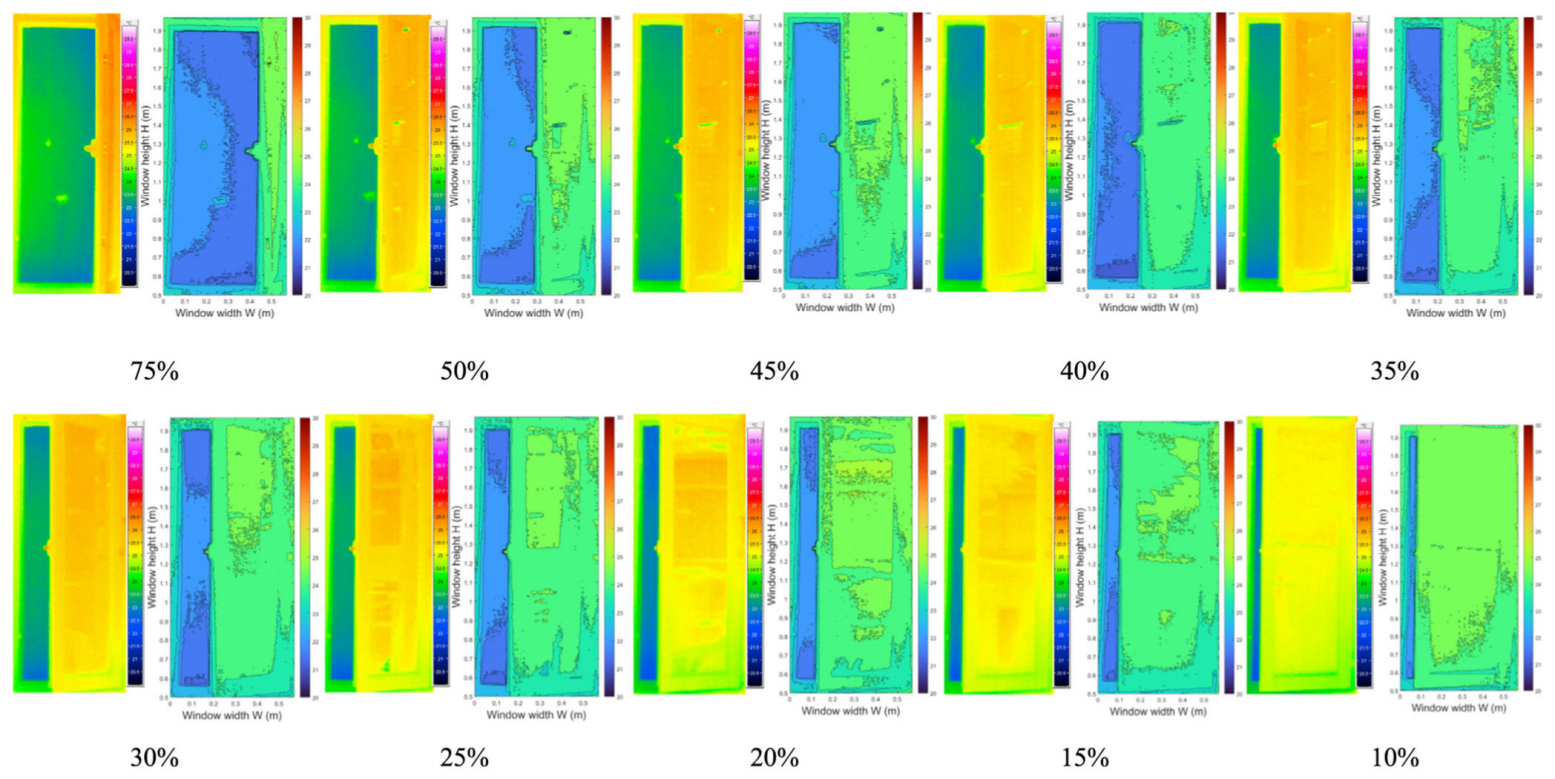

## Data Availability

Data will be made available on request.
